# Successful treatment of anastomotic leakage with an intestinal obstruction catheter and stent by colonoscopy: a case report and brief literature review

**DOI:** 10.3389/fonc.2024.1428452

**Published:** 2024-09-17

**Authors:** Wang Huang, Zhenzong Tan, Hao Sun

**Affiliations:** Department of Gastrointestinal Surgery, Chongqing University Cancer Hospital, Chongqing, China

**Keywords:** anastomotic leakage, colorectal cancer surgery, colonoscopy, intestinal obstruction catheter, intestinal stent

## Abstract

**Background:**

Anastomotic leakage (AL) is one of the most common, severe, and difficult-to-treat complications after colorectal cancer surgery. However, to date, the best treatment options for AL remain elusive.

**Case description:**

Here, we report the case of a 70-year-old man who had previously undergone Hartmann’s surgery and developed a large AL after a colostomy reversal surgery in an external hospital. The condition mainly manifested as passage of the fecal material through the abdominal drainage tube accompanied by fever after intestinal surgery. We used a new method involving a transanal obstruction catheter combined with an anastomotic stent, along with fasting, administration of parenteral nutrition, and anti-infection treatment. By following this approach, AL was successfully cured without any complications.

**Conclusion:**

To the best of our knowledge, this is the first case of the use of a transanal intestinal obstruction catheter combined with an anastomotic stent for treating colorectal AL; the findings may guide clinicians to better treat and manage AL.

## 1 Introduction

Colorectal cancer (CRC) is one of the most common malignant tumors, and nearly 90% of patients with CRC require tumor resection (1). The most common postoperative complications of CRC are surgical site infections, including wound infection, anastomotic leakage (AL), and abdominal infection, and the surgical site infection can be as high as 45% (2). AL is one of the most severe, lethal, and difficult-to-treat complications after CRC surgery. Intestinal AL refers to the condition of leakage of the internal intestinal fluid into the external colonic environment due to the defective integrity of the intestinal wall at the site of anastomosis. Abscesses near the anastomosis site should also be considered as leaks even if there is no apparent connection to the colonic cavity. AL is one of the most complications after colorectal surgery. The average incidence of AL after colon cancer surgery is 3.6% (3); however, the incidence of AL after rectal cancer surgery is as high as 27% (4). The mortality rate of colon cancer patients after AL is as high as 12% (5). Presently, there is no consensus on the best treatment option for AL. The management of AL should be individualized based on the patient’s general condition, the size and location of the anastomotic defect, the indication for primary resection, and the presence or absence of a proximal stoma. The treatment of AL often requires a combination of multiple approaches; consequently, it takes a long time and repeated surgeries to achieve cure. Here, we describe the case of a patient in whom an intestinal obstruction catheter combined with an anastomotic stent through the anus was used to successfully treat AL.

## 2 Case report

On May 2023, a 70-year-old man presented to our hospital with “13 days after colostomy reversal, abdominal drainage output for 7 days.” This patient is complicated with hypertension, which can be controlled by drugs. One year ago, the patient had undergone Hartmann’s surgery in an external hospital for treating acute intestinal obstruction. The postoperative pathological report revealed that the patient had moderately differentiated adenocarcinoma of the sigmoid colon; the tumor had invaded the outer intestinal wall, had a clear incisional margin, and no regional lymph nodes were involved (0/13). The postoperative pathological tumor stage was pT4aN0M0 IIB. No antitumor therapy was given at that time. During the postoperative period, the tumor did not recur or metastasize. Thirteen days ago, after any contraindication for surgery was excluded, laparoscopy was performed under general anesthesia, and the patient underwent a sigmoidostomy reversal operation in the external hospital. However, 6 days after the operation, excretion of fecal water into the drainage tube occurred, accompanied with malodors. The amount of the drainage fluid varied from 50 to 200 mL/day. Moreover, the patient repeatedly experienced fever during this period, with the highest temperature of 38.7°C. Treatment with an injection of piperacillin sodium and tazobactam sodium, fasting, negative pressure (a drainage connected to a suction collection bag) drainage, and other approaches were ineffective.

After examination, his ECOG (Eastern Cooperative Oncology Group) score was 1. On physical examination, a 15-cm healed midline laparotomy was found, and a 5-cm surgical incision (original stoma) was found in the left lower abdomen. Pus oozed out from the surface of the wound, and the depth of exploration was approximately 0.5 cm. A drainage tube was detected in the right abdomen. Leakage of fecal material from the tube was observed, accompanied with a bad odor; the volume of the fecal material was approximately 20 mL. The lower abdomen was soft, with deep tenderness and no rebound abdominal pain. The frequency of bowel sounds was 3–5 times/min.

The findings of laboratory examination after admission were as follows: The blood routine and C-reactive protein (CRP) are shown in **Table 1**. Other laboratories were generally normal during hospitalization. Bacterial culture of the drainage fluid was positive for Escherichia coli and Enterococcus faecium. There were no CT and other imaging reports from other hospitals. The body temperature, drainage fluid, blood routine and CRP during hospitalization are shown in **Table 1**.

**Table 1:** W stands for white blood cell, normal value is 3.5-9.5x109/L; N is for neutrophils, normal value 1.8-6.3x109/L.

Timing OF Interventions(Date)	1	5	9	14	24
Interventions	Intestinal obstruction catheter and stent by colonoscopy; Anti-infective Therapy; Parenteral nutrition support; Continuous irrigation(500 mL/d);	Enteral nutrition; Anti-infective therapy was suspended; Continuous irrigation(500 mL/d);	Colonoscopy was repeated; Continuous irrigation(500 mL/d);	Replaced the stent and Intestinal obstruction catheter; Removed the abdominal drainage tube; Continuous irrigation(500 mL/d);	Removed the stent and intestinal obstruction catheter; Cured the anastomotic fistula; Continuous irrigation(500 mL/d);
Drainage fluid (ml)	5	0	0	-	-
Temperature (Tmax °C)	38.3	normal	normal	normal	normal
Blood routine examination (× 109/)	W 10.09; N 8.82;	W (normal) N 7.38;	normal	normal	normal
CRP (mg/L)	90.36	50.96	normal	normal	normal

The normal value of CRP was 0-10mg/L.

**Figure 1:**
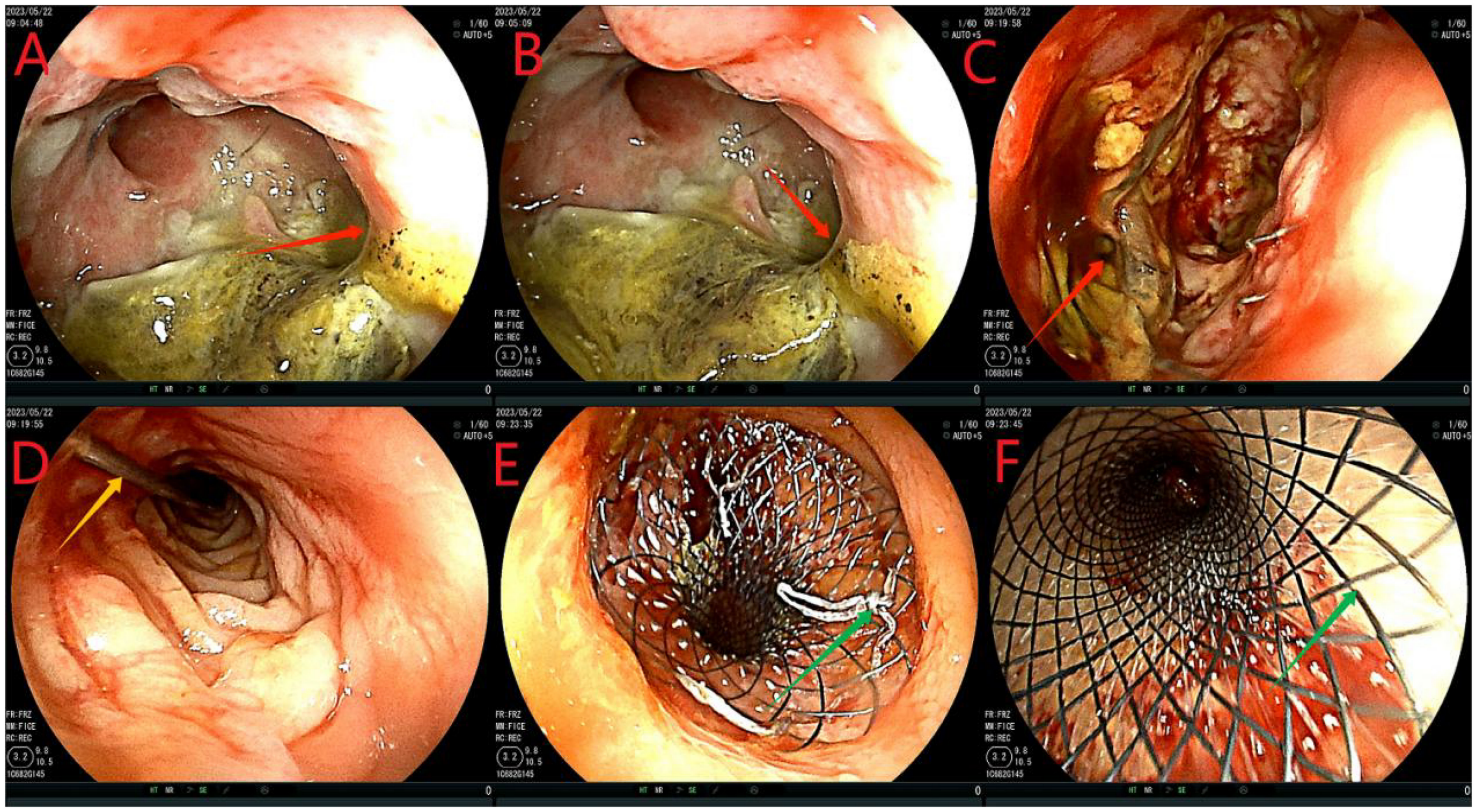
Flow chart of stent implantation. **(A-C)**, red arrows indicate anastomotic fistula; **(D)**, yellow arrows indicate the stent guidewire; **(E, F)** denote the release of the stent under colonoscopy. This stent is a nickel-titanium memory alloy self-expanding stent, which is modified from a nickel-titanium memory alloy esophageal stent and is a coated stent. It is 10cm in length with a diameter of 32 to 35mm on the proximal port side and 28 to 30mm on the distal anal side.

**Figure 2:**
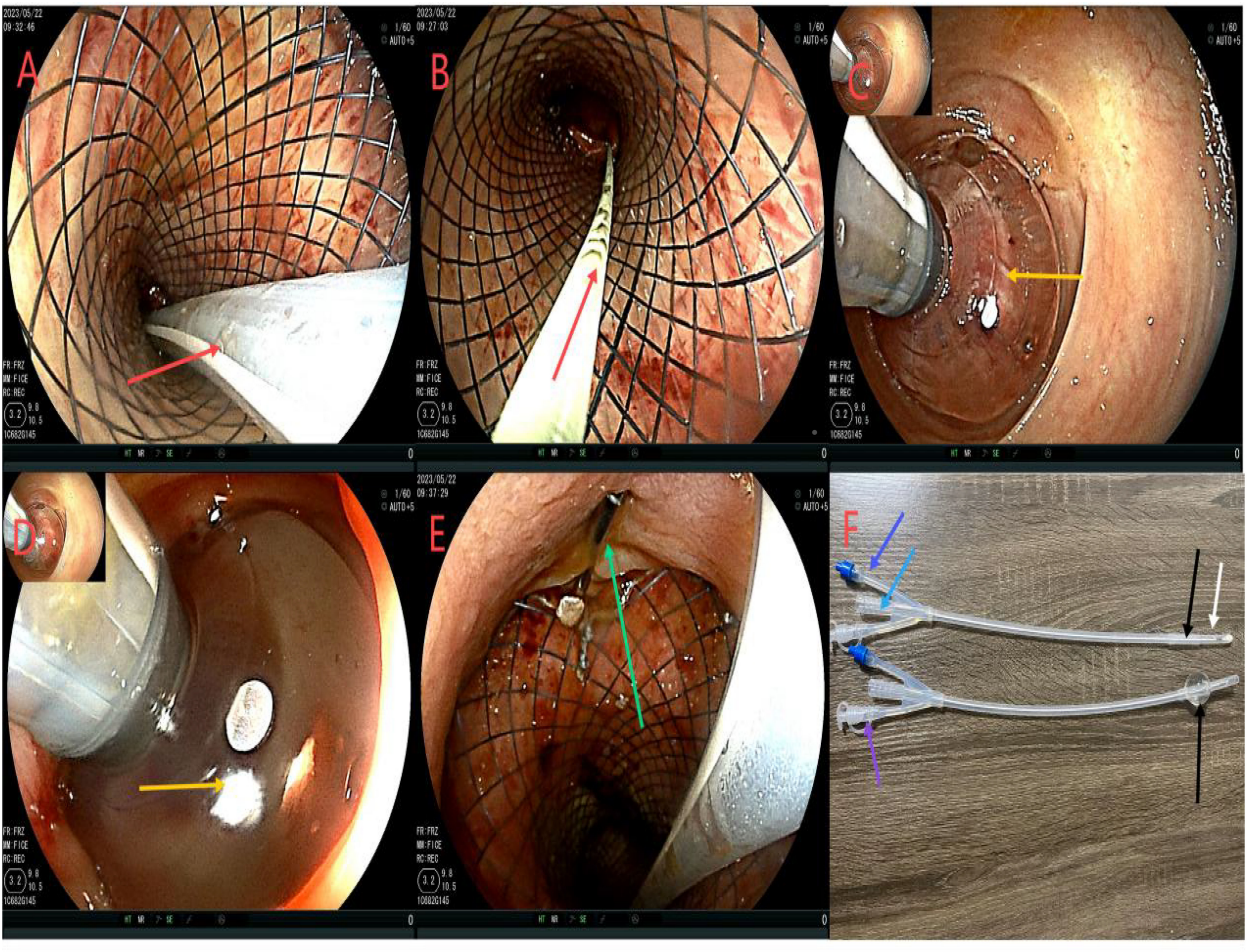
Flowchart of ileus catheter insertion. **(A, B)**, the red arrow refers to ileus catheter insertion under the colonoscope; **(C, D)**, the yellow arrow shows the ileus catheter injection of a 15 mL water sac to fix the ileus catheter to avoid slippage; **(E)**, the green arrow shows stent fixation with 3 titanium clips at the distal end to prevent slipping of the stent. **(F)**, is the modified intestinal obstruction catheter, and the material is the intestinal obstruction catheter material. It is a three-chamber capsule catheter. The length is 50cm, and the purple arrow indicates the flushing tube (internal diameter of 3mm), which is continuously flushed; The sky blue arrow indicates the drained stool (model 24Fr, 8 mm in diameter), which can be connected to the outside of the urine bag. The sky blue color arrow is the water injection tube (5 to 25 mL of normal saline can be injected according to the size of the intestinal wall). However, the black color of the tip refers to the position of the water sac, which is the water sac filled with water and the water sac without water, and the white color is the side hole of the tip, which can avoid the obstruction of the lumen by stool and enhance drainage. While there is a white protective cap at the tip, which is removed when used.

After admission, based on the results of drug sensitivity tests conducted in other hospitals, the patient was treated with piperacillin-tazobactam (4.5 g q8h), together with fasting and parenteral nutrition. Transanal enema was performed immediately after admission (May 22, 2023), followed by colonoscopy for anastomotic stent implantation and transanal catheter implantation for anorectal obstruction. The specific steps were as follows. First, the anastomosis site was found 8 cm away from the anus. A leak of approximately 1.5 cm was detected at 10-1 o’clock at the lithotomy position (red arrows in **Figures 1A, B**). The anastomosis site and the intestinal cavity were rinsed repeatedly, and a necrotic tissue was observed (red arrows in **Figures 1B, C**). By using an endoscope, a guidewire was passed to the proximal end of the anastomotic mouth (**Figure 1D**, the yellow arrow represents the stent guidewire). Subsequently, a nickel-titanium memory alloy self-expanding stent was placed, with the proximal and distal ends of the stent at approximately 15 and 5 cm from the anus, respectively (**Figures 1E, F**). An endoscopically guided transanal obstruction catheter (**Figure 2F**) of approximately 20 cm was then inserted (**Figures 2A, B**, the red arrow indicates the intestinal obstruction catheter), followed by injection of a 15 mL balloon water to fix the catheter (**Figures 2C, D**). The distal end of the stent was fixed with three titanium clips (**Figure 2E**, green arrow). Finally, the intestinal obstruction catheter was fixed to the skin of the anal border.

**Figure 3:**
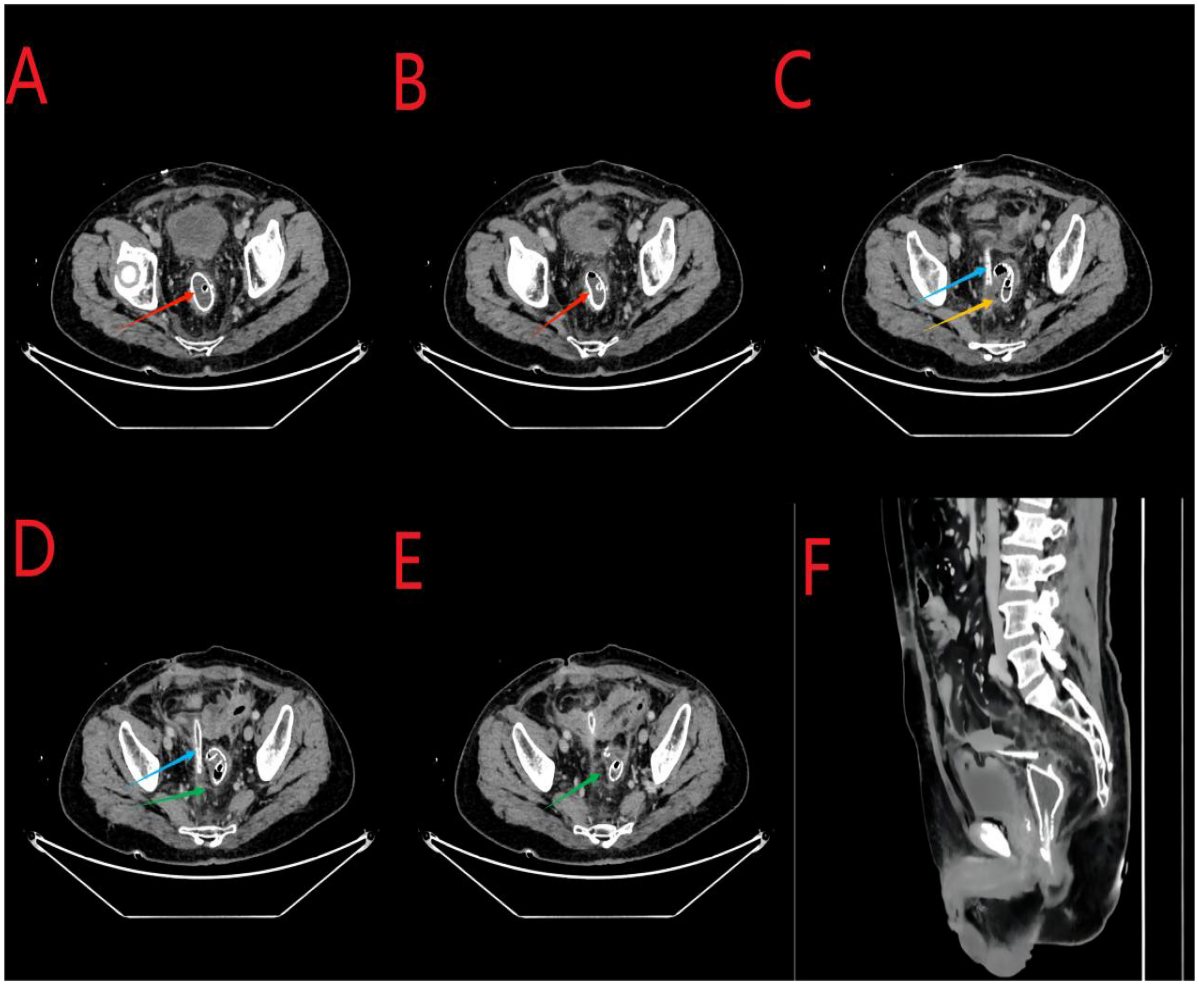
**(A, B)**, the red arrows indicate the stent at the anastomosis site. **(C)**, the yellow arrow indicates air accumulation around the anastomosis site. **(C, D)**, the sky-blue arrows in the middle indicate the abdominal drainage tube. **(D, E)**, the green arrows indicate excessive exudation in the pelvic cavity with mesenterial thickening. **(F)**, the arrows indicate the sagittal position.

An abdominal enhanced CT revealed the following findings. The wall of the anastomosis site was thickened and rough, and there was little fluid and gas accumulation near the right side of the anastomosis site; this observation was consistent with the clinical manifestations of AL. The pelvic cavity showed more exudation, with the thickening of the peritoneum and mesangium (**Figure 3**).

**Figure 4:**
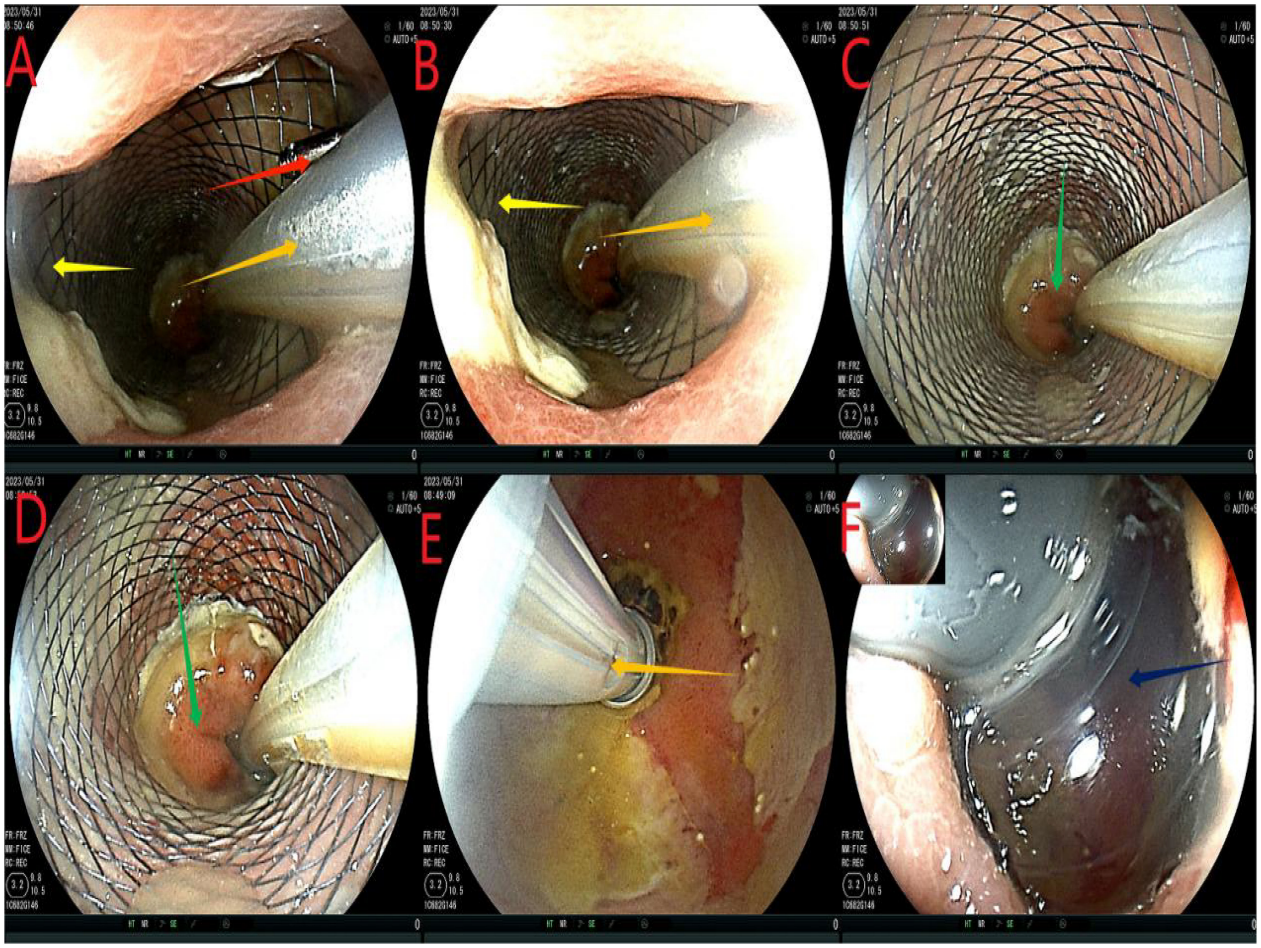
Reexamination of colonoscopy. **(A, B)**, the red arrow refers to the titanium clip under the intestinal stent, the orange arrow indicates the intestinal obstruction catheter, and the yellow arrow shows the anastomotic stent. **(C, D)**, the green arrows show the intestinal mucosa proximal to the scaffold. **(E)** Yellow is the ileus catheter, draining the stool. **(F)**, the dark blue color represents the water sac of the intestinal obstruction catheter. No significant fecal residue was detected in the intestinal cavity distal to the obstruction water sac.

On the first day after the intervention, the abdominal drainage of the patient was reduced to 5 mL, and no change in abdominal signs and no fever were observed. However, the patient complained of anal distension and received analgesic treatment. Additionally, 500 mL of sodium chloride solution was injected into the intestine through the intestinal obstruction catheter, and the remaining treatment was the same as that given before. On the 5th day after the intervention, the abdominal signs of the patient completely disappeared, the drainage fluid was reduced to 0 ml (**Table 1**). Reexamination of blood routine and C-reactive protein (CRP) are shown in **Table 1**; Other laboratory tests showed no significant abnormalities. Treatment with piperacillin-tazobactam was discontinued, and the patient began to resume enteral nutrition, complete fluids such as enteral nutritional powder, with a gradual reduction in parenteral nutrition. Nine days after the intervention, a colonoscopy re-examination revealed no displacement of the stent or ileus catheter (**Figure 4**). The drainage was 0 mL during this period.

**Figure 5:**
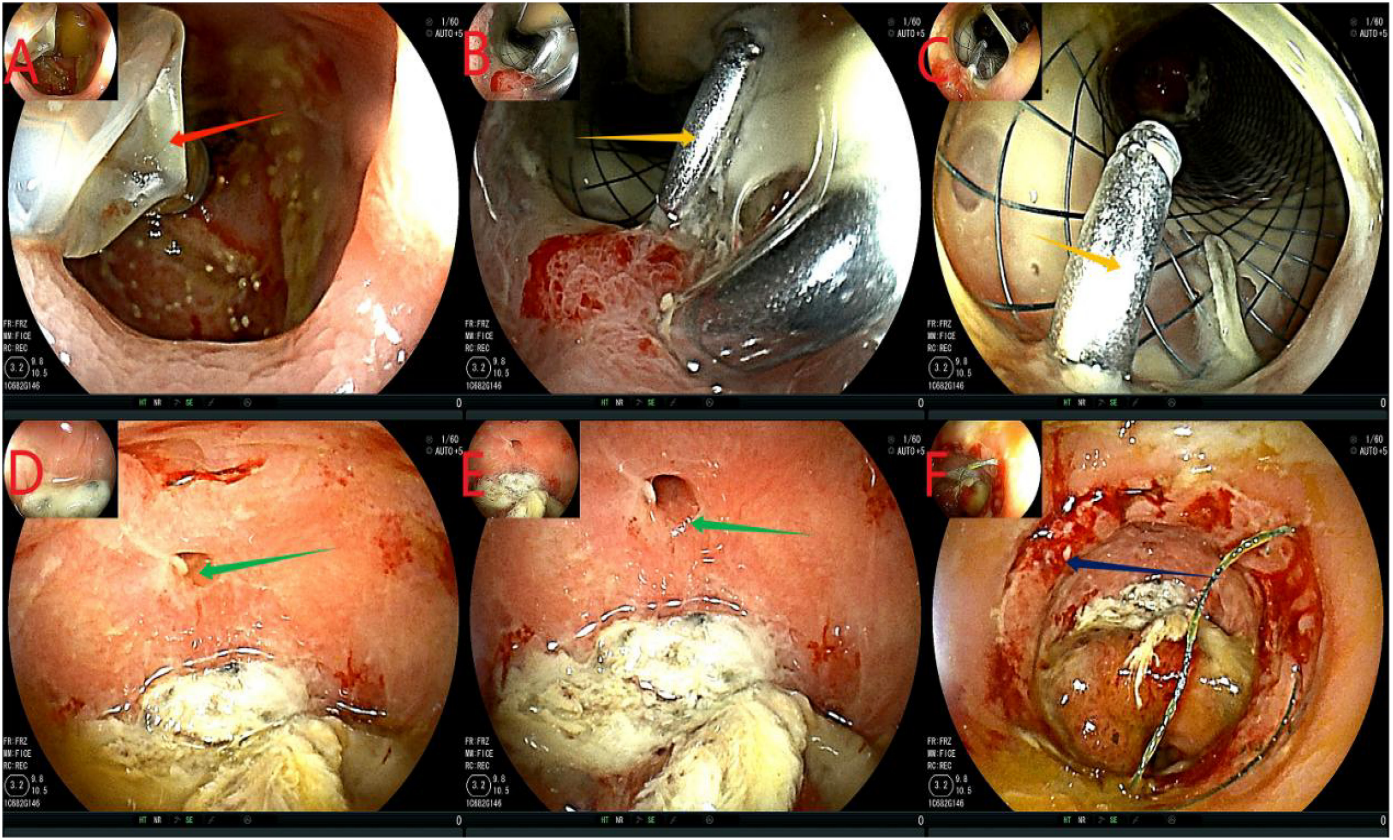
Flowchart of removing the ileus catheter and stent. **(A)**, the red arrow indicates extracting water from the effluent sac and removing the ileus catheter. **(B, C)**, the orange arrows show the titanium clips below the support. **(D, E)**, the green arrows indicate the anastomotic leakage, which was smaller than before, with a smooth mucosa. Panel **(F)** shows the anastomosis in dark blue.

Fourteen days after the intervention, we removed the ileus catheter and stent. First, we confirmed that there was no displacement of the stent or the ileus catheter (similar to **Figure 4**). Next, the ileus catheter was removed (**Figure 5A**). All clips were then removed endoscopically (**Figure 5B**). Subsequently, the stent was tightened and removed successfully. Finally, at the 11 o’clock position of the anastomosis site (lithotomy position), a leakage of approximately 0.5 cm was detected, which was significantly smaller than that observed before; the surrounding mucosa was smooth (**Figures 5D, E**). The anastomotic stent and the transanal intestinal obstruction catheter were placed again according to the previous steps (**Figures 1**, **2**). The abdominal drainage tube was removed on the same day. Anal irrigation, enteral nutrition support, and analgesic treatment were continued. During the period, the drainage was 0 mL, and the patient had no abdominal signs.

**Figure 6:**
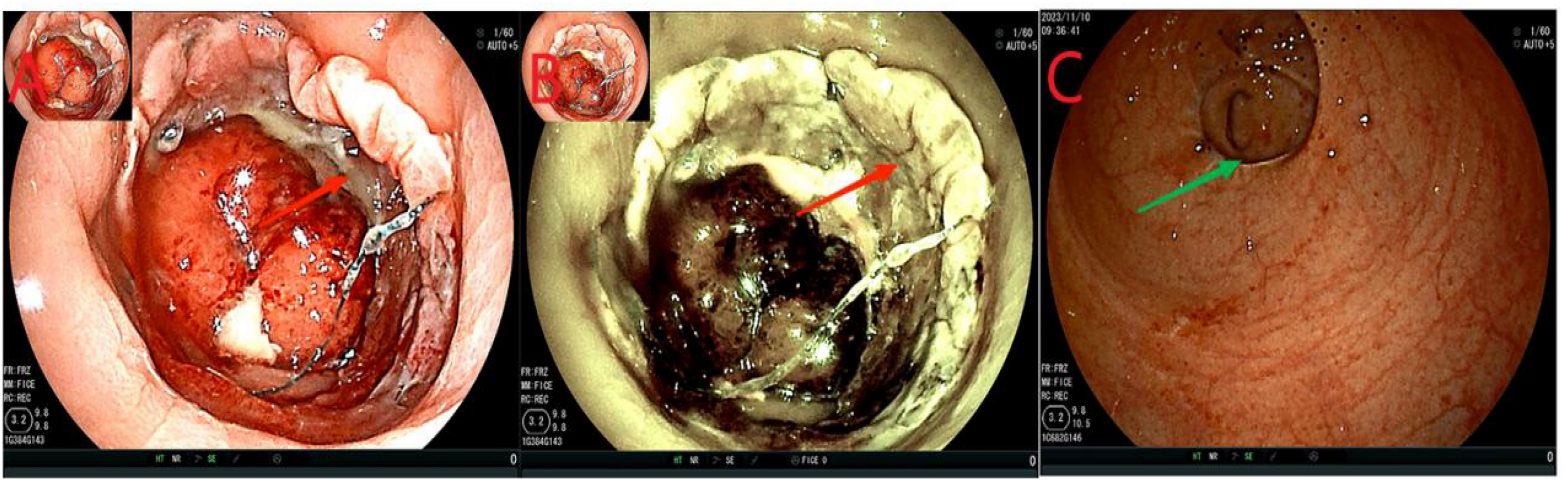
**(A, B)**, the anastomosis site indicated by the red arrows showed intact smooth mucosa with visible granulomas and no leakage. **(C)**, the green arrow indicates the anastomosis.

**Figure 7:**
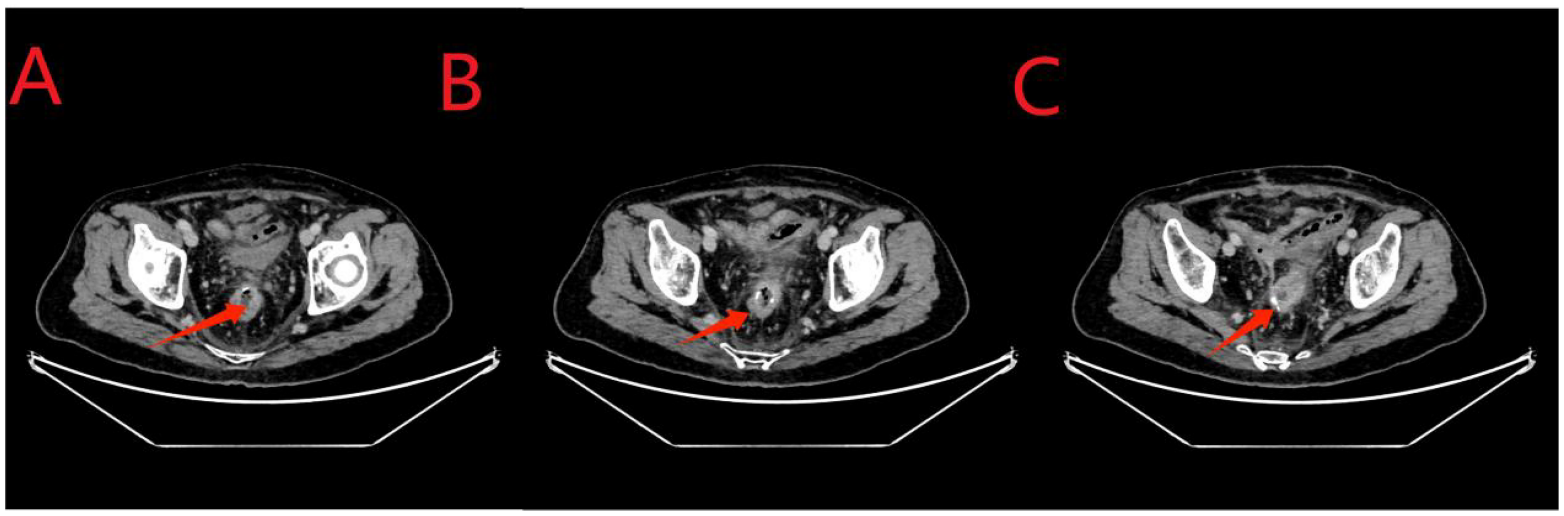
**(A-C)**, the red arrows indicate the anastomosis. Compared to the results obtained for the examination on May 22, 2023, no fluid or gas accumulation was observed, and the seepage was significantly reduced.

Twenty-four days after the intervention, a repeat colonoscopy was performed, and the stent and the transanal intestinal obstruction catheter were removed (**Figure 5** in sequence). The anastomosis site showed good healing, and the surrounding mucosa was reddish (**Figure 6**). A CT scan revealed no anastomotic abnormalities (**Figure 7**). The patient’s entire treatment course is summarized in **Table 1**. The patient was discharged 25 days after the procedure, with no adverse events. After discharge, the patient defecated 2–3 times a day and did not complain of discomfort such as anal distension, frequent defecation, or urgency of defecation. More than 5 months after the intervention, a review colonoscopy was performed, and the anastomosis site was found to be smooth and complete, as shown in **Figure 6C**.

## 3 Discussion

The incidence and mortality rates of CRC are increasing each year. Although various treatment methods for CRC are available, surgery remains the primary choice for comprehensive treatment. However, despite the improvement in surgical techniques, suturing methods, and surgical equipment, the occurrence of AL still cannot be avoided. According to a study conducted by a large registry, the AL rate after rectal cancer surgery in Germany and Sweden was 10.8% (6) and 11.9% (7), respectively. Moreover, in the TaTME registry, the AL rate was reported as 15.7% (8) or even as high as 29.2% (9). Studies have shown that male sex, BMI, obesity, coexisting pulmonary disease, anesthesia ASA score, emergency surgery, open surgery and type of resection were risk factors for anastomotic leakage after colon cancer surgery (3). Studies have shown that compared with laparoscopy, robot can significantly reduce anastomotic leakage after colorectal cancer surgery (10, 11), and even 0% anastomotic leakage (12),. The occurrence of AL not only increased the rate of reoperation and antibiotic use, but it also reduced the quality of life of patients through increased rate of ostomy and increased hospitalization cost and perioperative mortality rate. It even resulted in negative outcomes for patients with tumors (increased local recurrence rate and decreased overall survival rate (13–15).

Many treatment methods are available for anastomotic fistula; however, there is yet no consensus on the best treatment option. This might be because AL is a small and complex disease, and there is a lack of a large number of prospective and retrospective studies. AL can be cured through surgical and nonsurgical treatments. Surgical treatment for patients with apparent suppurative or fecal peritonitis or unstable vital signs ranges from simple fecal diversion, resection of the anastomosis site, and re-anastomosis (with a reoperation leakage rate of up to 41% (16) to dissection of the anastomosis site (with Hartmann’s surgery, up to approximately 50% for permanent ostomy) (17). Nonsurgical treatments include antibiotic use (18), local drainage (19), nutritional support, and endoscopic treatment (18). In the present case, there was no suppurative or fecal peritonitis after the occurrence of AL, and the patient’s vital signs were stable. Conservative treatment was applied in the external hospitals and our hospital. The patient was treated with antibiotics, local drainage, and nutritional support for 1 week. However, the best results were not achieved, and healing of the anastomotic site was not observed.

In recent years, new treatment options for AL have emerged, including internal sponge treatment [cure rate: 73–79% (20, 21)], endoscopic clamping [cure rate: 60.2–86% (22, 23)], stent placement [cure rate: 86% (24)], endoscopic vacuum treatment [EVT, cure rate: 70–85.3% (25, 26)], and surgical endoscopic vacuum-assisted closure [cure rate: 82% (27)]. However, the endoscopic treatment of AL has been a single-center study with small sample size, and the best treatment has not reached a consensus. However, after continuous research, the above technology is relatively mature. When negative vacuum systems are not available or endoscopic technology is not mature enough, our protocol can be tried. Moreover, endoscopic treatment of AL is mostly performed under the proximal stoma, which improves only the healing rate of AL but does not reduce the stoma rate; this causes the patients to still face the risk of permanent stoma. Our protocol, compared with other treatment options for AL, our protocol can avoid a proximal stoma.

Previous studies have shown that the placement of anastomotic stents after rectal cancer surgery can reduce the occurrence of AL (28). In the present case, the anastomotic stent bypassed the effective space between the leakage mouth and the pelvic cavity and remained attached to the granulation tissue to cover the leakage mouth, thus achieving an effect equivalent to that of EVT. The incidence of stent displacement is as high as 58% (28). We placed three titanium clamps on the distal intestinal tube clamp to prevent intestinal peristalsis from slipping or displacing the stent. Studies have shown that repeated anal irrigation (29, 30) and repeated irrigation and aspiration are beneficial to anastomotic healing (31). There are, however, few reports on the use of continuous lavage and endoscopic lavage for treating anastomotic fistula. Fortunately, our present case report fills this gap. Moreover, the intestinal obstruction catheter is a new type of three-lumen guide modified by us, in which 5–25 mL water capsule is injected into the water sac to prevent slipping from the intestinal cavity. The middle tube was 24Fr, 8mm in diameter, and was used for defecation; This approach is equivalent to enterostomy in that it keeps the anastomosis site relatively clean and allows the patient to resume enteral nutrition as soon as possible, thus avoiding two operations of stoma and stoma back. In addition, a small tube (3mm inner diameter) was used for slow saline infusion to prevent fecal obstruction of the catheter. The large diameter and short length of the anal obstruction catheter facilitated adequate drainage of faeces, thus facilitating patient care.

## 4 Conclusion

AL is an inevitable complication after colon surgery, and there is yet no consensus on the best treatment approach for AL. To the best of our knowledge, the present case is the first in which an intestinal obstruction catheter combined with an anastomotic stent was inserted through the anus to successfully cure AL, thus avoiding surgeries related to stoma creation and stoma reversal. However, our proposed approach needs more clinical data to confirm its safety, effectiveness, and feasibility.

## Data Availability

The original contributions presented in the study are included in the article/Supplementary Material. Further inquiries can be directed to the corresponding author.
